# The Functional Role of Myogenin in Cardiomyoblast H9c2 Cells Treated with High Glucose and Palmitic Acid: Insights into No-Rejection Heart Transplantation

**DOI:** 10.3390/ijms241713031

**Published:** 2023-08-22

**Authors:** Po-Shun Hsu, Shu-Ting Liu, Yi-Lin Chiu, Chien-Sung Tsai

**Affiliations:** 1 Graduate Institute of Medical Sciences, National Defense Medical Center, Taipei 114, Taiwan; hsuposhun@gmail.com; 2 Division of Cardiovascular Surgery, Department of Surgery, Tri-Service General Hospital, National Defense Medical Center, Taipei 114, Taiwan; 3 Department of Biochemistry, National Defense Medical Center, Taipei 114, Taiwan; shuting0719@gmail.com (S.-T.L.); yilin1107@gmail.com (Y.-L.C.)

**Keywords:** myogenin, palmitic acid, cell differentiation, reactive oxygen species, transplantation

## Abstract

Various pathological alterations, including lipid-deposition-induced comparative cardiac lipotoxicity, contribute to cardiac aging in the failing heart. A decline in endogenous myogenin proteins can lead to the reversal of muscle cell differentiation and the creation of mononucleated muscle cells. Myogenin may be a specific regulator of adaptive responses to avoid pathological hypertrophy in the heart. Hence, it is important to understand the regulation of myogenin expression and functions in response to exposure to varied stresses. In this study, we first examined and verified the cytotoxic effect of palmitic acid on H9c2 cells. The reduction in *myogenin* mRNA and protein expression by palmitic acid was independent of the effect of glucose. Meanwhile, the induction of *cyclooxygenase 2* and *activating transcription factor 3* mRNAs and proteins by palmitic acid was dependent on the presence of glucose. In addition, palmitic acid failed to disrupt cell cycle progression when H9c2 cells were treated with no glucose. Next, we examined the functional role of myogenin in palmitic-acid-treated H9c2 cells and found that myogenin may be involved in palmitic-acid-induced mitochondrial and cytosolic ROS generation, cellular senescence, and mitochondrial membrane potential. Finally, the GSE150059 dataset was deposited in the Gene Expression Omnibus website and the dataset was further analyzed via the molecular microscope diagnostic system (MMDx), demonstrating that many heart transplant biopsies currently diagnosed as no rejection have mild molecular-antibody-mediated rejection-related changes. Our data show that the expression levels of *myogenin* were lower than the average level in the studied population. Combining these results, we uncover part of the functional role of myogenin in lipid- and glucose-induced cardiac cell stresses. This finding provides valuable insight into the differential role of fatty-acid-associated gene expression in cardiovascular tissues. Additionally, the question of whether this gene expression is regulated by myogenin also highlights the usefulness of a platform such as MMDx-Heart and can help elucidate the functional role of myogenin in heart transplantation.

## 1. Introduction

The heart is the organ with the greatest caloric needs and the most robust oxidation of fatty acids. Heart aging is accompanied by several pathological changes, including lipid-deposition-induced cardiac lipotoxicity [1]. Exceeding the capacity for storage and oxidation of intramyocardial lipids induces non-ischemic and non-hypertensive cardiomyopathy, termed lipotoxic cardiomyopathy [2]. Anti-diabetic agents and lowering cholesterol statins have no beneficial effect on outcomes in heart failure (HF) patients without atherosclerotic diseases [3]. The pathogenesis of their cardiomyopathy is multifactorial, and it is still a challenge to identify the trigger for cellular signaling and the modification of proteins and lipids in diabetic cardiomyopathy.

Lipid-induced apoptosis, ceramide accumulation, endoplasmic reticulum (ER) stress, mitochondrial dysfunction, and reactive oxygen species (ROS) overproduction might play individual and independent roles in the pathogenesis of lipotoxic cardiomyopathy. Hence, there has been more attention paid to the effect of dietary fat intake on the progression of cardiovascular disease and HF in recent years [4,5,6]. In cardiomyocytes, palmitate is one of the saturated fatty acids that has been demonstrated to be more lipotoxic than unsaturated fatty acids [7,8]. ER stress is the major mechanism underlying palmitate-induced cardiomyocyte dysfunction for the induction of cell death [9,10]. It is well known that exposure to saturated fatty acids but not to unsaturated fatty acids induces cellular apoptosis in cell culture. The H9c2 cell line, a subclone of a cell line derived from embryonic BD1X rat heart tissue, exhibits many of the properties of skeletal muscle [11]. H9c2 cells differentiate from myoblasts into myotubes when cultured in lower-serum conditions and/or treated with all-trans retinoic acid [12]. Although H9c2 cells share some characteristics of differentiated cardiac cells, they lack the typical organization and dense packing of adult cardiomyocytes [11,13]. Palmitate treatment induced the release of cytochrome c and mitochondrial dysfunction precede ceramide accumulation in primary neonatal cardiac myocytes [14]. Ceramide treatment is sufficient to induce apoptosis, palmitate was found to increase cellular ROS accumulation, and ER stress preceded apoptosis in H9c2 cells. However, the contribution of ROS versus lipids and the source of ROS generation remain to be determined in H9c2 cells.

Muscles are composed of different fiber types based on their specific myosin heavy chain (MHC) isoforms to fulfill various functional needs [15]. However, the regulation of skeletal-muscle-specific MHC expression in the developing myocardium remains to be investigated. The capacity of 5-azacytidine to induce human mesenchymal stem cells to differentiate into both cardiomyocytes and skeletal myocytes has been demonstrated [16]. Myogenin was found to ameliorate Ang-II-induced apoptosis of human-induced pluripotent-stem-cell-derived cardiomyocytes by downregulating the expression of proinflammatory genes via an analysis of RNA sequencing data [17]. Myogenin, MyoD, Myf5, and MRF4 are members of the MyoD family of transcription factors for muscle differentiation [18,19]. Myogenin is a muscle-specific basic helix-loop-helix transcription factor, and its activity is a turning point for irreversible commitment to terminal differentiation [20,21]. Myogenin expression and skeletal muscle differentiation are preceded by the downregulation of cAMP content in decreasing adenylate cyclase activity. In H9c2 cells, the skeletal muscle differentiation program can be inhibited via the pharmacological increase of intracellular cAMP content.

The maintenance of skeletal muscle structure and function requires innervation by motor neurons. Myogenin plays an important role in the balance of terminal muscle cell differentiation and differentiated muscle cells may be dedifferentiated through its downregulation [20]. Many factors, including HuR, Twist, CACNb1 (voltage-gated calcium channel β1), *miR-186*, and *miR-328*, are involved in the regulation of *myogenin* gene expression [22,23,24,25,26,27]. Myogenin and the forkhead box O (Foxo) family of proteins are transcription factors for the regulation of the expression of the E3 ubiquitin ligases, muscle RING finger 1 (MuRF1) and muscle atrophy F-box/atrogin-1, to control protein degradation and muscle atrophy [28]. A recent study showed that myogenin may be a specific regulator of adaptive responses and Foxo1 and 3a may primarily regulate other processes such as antioxidant response to avoid pathological hypertrophy in the heart [29]. One study showed that forced expression of myogenin in the skeletal muscle of histone deacetylase mutant mice restores muscle atrophy following denervation, suggesting that myogenin is a specific potential therapeutic target for muscle wasting. The reduction mechanisms of myogenin during HF need further investigation.

Recent studies demonstrated that *myogenin* might be a target for doxorubicin-induced cardiotoxicity and high-Pi-suppressed myogenic differentiation [27,30]. H9c2 cells treated with palmitate displayed increased cellular ROS accumulation and ER stress preceding apoptosis [31]. In this study, we sought to investigate the functional role of myogenin in palmitate induced H9c2 cell death. Hence, we designed experiments to address the relationship of glucose and palmitate concentrations to the expression of *myogenin*. Then, we addressed the functional role of myogenin in various cellular stresses, including lipotoxicity, in H9c2 cells. Combined with these results, we provide some insights into the functional role of myogenin in lipid- and glucose-induced cardiac cell stresses. Our work also sheds light on the understanding of myogenic factors involved in aging heart functions.

## 2. Results

### 2.1. The Effects of Palmitic Acid Were Examined in H9c2 Cells Cultured with and without Glucose

**Figure 1:**
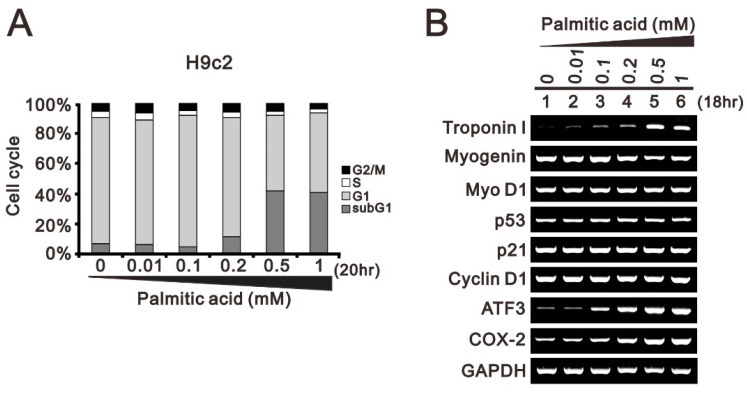
The effects of palmitic acid on cell cycle profile and mRNA gene expression in H9c2 cells. H9c2 cells were treated with the indicated concentrations of palmitic acid (0, 0,01, 0.1, 0.2, 0.5, 1 mM) for 20 h or 18 h. (**A**) After treatment, the cells were stained with PI dye and then subjected to flow cytometry analysis to measure cell cycle profiles. (**B**) Total RNA was subjected to RT-PCR analysis. *GAPDH* was used as a loading control.

Hypertriglyceridemia implies increased plasma levels of fatty acids, which are taken up and stored in lipid droplets in the heart. Palmitic acid, a saturated fatty acid, is more lipotoxic than unsaturated fatty acids in cardiomyocytes [4,6]. In this study, we first treated H9c2 cells with concentrations of palmitic acid up to 1 mM for 20 h. Our cell cycle profile shows that palmitic acid induced the sub-G1 phase and suppressed the G1, S, and G2/M phases in H9C2 cells, indicated by corresponding changes in population (Figure 1A). Our RT-PCR analysis *Troponin I* mRNA was induced, *myogenin* and the *MyoD* mRNAs were slightly suppressed, and *p53*, *cyclin D1*, and *p21* mRNAs remained constant after treatment with palmitic acid in H9C2 cells (Figure 1B). The gene expressions of *activating transcription factor 3* (*ATF3*) and *cyclooxygenase 2* (*COX-2*) were induced in a dose-dependent manner.

**Figure 2:**
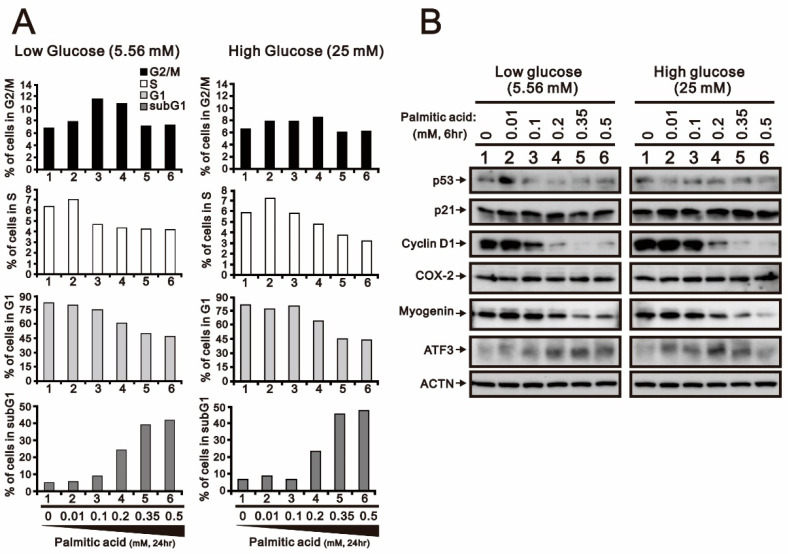
The effects of low- and high-glucose conditions combined with palmitic acid on cell cycle profile and protein expression in H9c2 cells. H9c2 cells were cultured in 5.56 mM low-glucose and 25 mM high-glucose conditions and treated with the indicated concentrations of palmitic acid (0, 0.01, 0.1, 0.2, 0.35, 0.5 mM) for 24 h or 6 h. (**A**) After treatment, the cells stained with PI dye and then subjected to flow cytometry analysis to measure cell cycle profiles. (**B**) Cell lysates were subjected to Western blotting analysis. ACTN was used as a loading control protein.

We further examined the effect of glucose concentration on the palmitic-acid-induced sub-G1 populations. Compared with 5.5 mM and 25 mM glucose, palmitic acid induced the sub-G1 phase and suppressed the G1 and S phases, as indicated by corresponding changes in population (Figure 2A). The populations of the G2/M phases were first increased and then returned to the basal level in response to palmitic acid under both concentrations of glucose. Decreasing amounts of myogenin protein and increasing amounts of ATF3 in response to palmitic acid were consistently observed in H9c2 cells (Figure 2B). However, p53, cyclin D1, and COX-2 proteins had inconsistent expression patterns, observed via their mRNAs.

**Figure 3:**
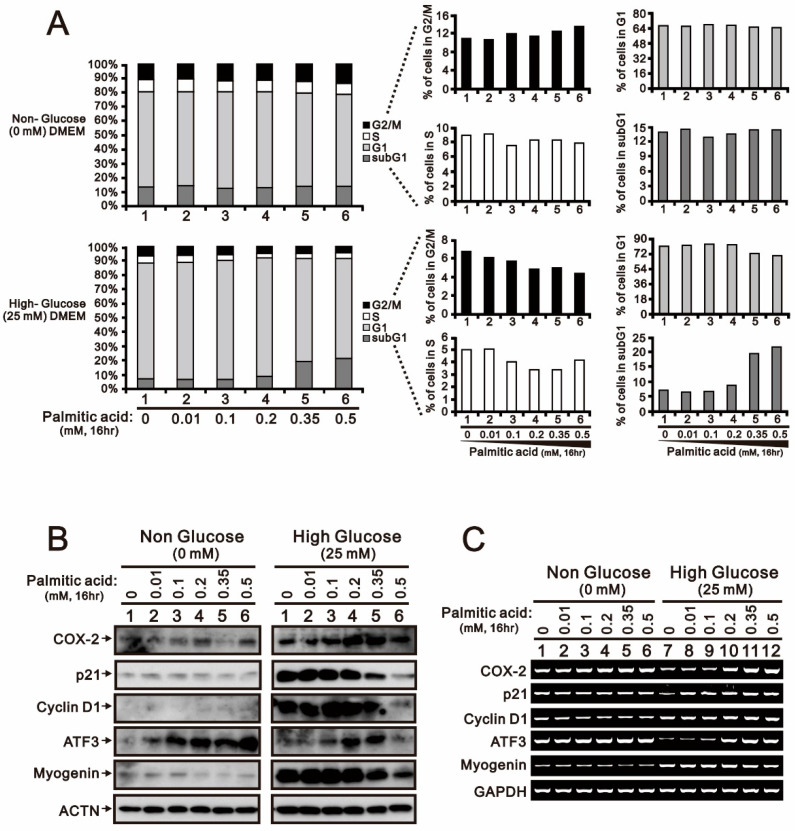
The effects of non- and high-glucose conditions combined with palmitic acid on cell cycle profile, proteins, and mRNA gene expression profile in H9c2 cells. H9c2 cells were cultured in 0 mM non-glucose and 25 mM high-glucose conditions and treated with the indicated concentrations of palmitic acid (0, 0.01, 0.1, 0.2, 0.35, 0.5 mM) for 16 h. (**A**) After treatment, the cells stained with PI dye and then subjected to flow cytometry analysis to measure cell cycle profiles. (**B**) Cell lysates were subjected to Western blotting analysis. ACTN was used as a loading control protein. (**C**) Total RNA was subjected to RT-PCR analysis. *GAPDH* was used as a loading control.

We failed to observe the effect of palmitic acid on the cell cycle profile, including the sub-G1 phase, when H9c2 cells were cultured in the absence of glucose (Figure 3A). Palmitic acid induced the sub-G1 phase and suppressed the G1, S, and G2/M phases in H9C2 cells cultured with 25 mM glucose, as evidenced by corresponding changes in population. The basal levels of COX-2, p21, cyclin D1, and myogenin proteins in 25 mM glucose-cultured H9c2 cells were higher than those in no-glucose-treated H9c2 cells (Figure 3B). The basal levels of *COX-2*, *p21*, and *ATF3* mRNAs in no-glucose-cultured H9c2 cells were higher than those in 25 mM glucose-treated H9c2 cells (Figure 3C).

### 2.2. Evaluating the Functional Roles of Myogenin in Palmitic-Acid-Treated H9c2 Cells

**Figure 4:**
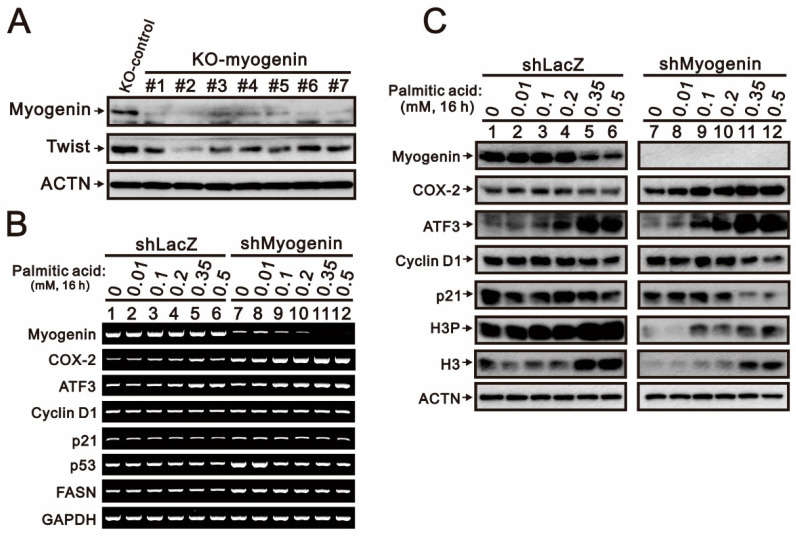
The effects of *myogenin* gene silencing combined with palmitic acid on the mRNA gene expression profile and proteins in H9c2 cells. (**A**) The *myogenin* gene in H9c2 cells was knocked down using 7 short-hairpin *myogenin* (*shMyogenin*) constructs, and *shLacZ* was used as the silencing control. The *shLacZ* and *shMyogenin* (#2) cells were treated with the indicated concentrations of palmitic acid (0, 0.01, 0.1, 0.2, 0.35, 0.5 mM) for 16 h. (**B**) Total RNA was subjected to RT-PCR analysis. *GAPDH* was used as a loading control. (**C**) Cell lysates were subjected to Western blotting analysis. ACTN was used as a loading control protein.

**Figure 5:**
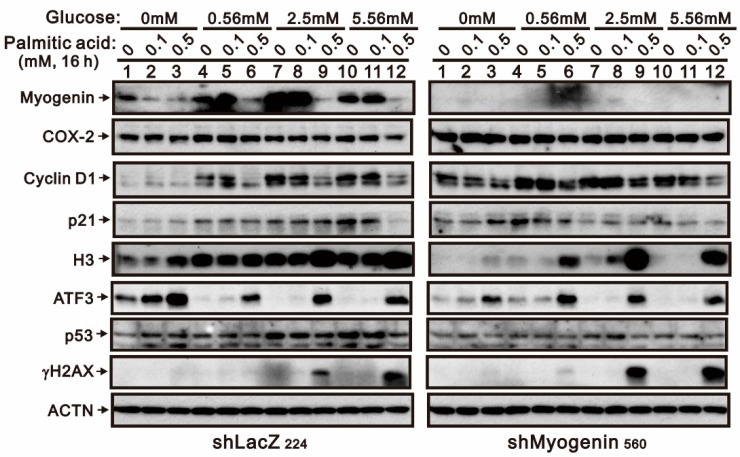
The effects of *myogenin* gene silencing in different glucose culture conditions combined with palmitic acid on the protein expression profile in H9c2 cells. The *shLacZ* and *shMyogenin* H9c2 cells were cultured in 0, 0.56, 2.5, and 5.56 mM glucose conditions and treated with the indicated concentrations of palmitic acid (0, 0.1, 0.5 mM) for 16 h. Cell lysates were subjected to Western blotting analysis. ACTN was used as a loading control protein.

The suppression of *myogenin* mRNA and protein was consistently affected by palmitic acid in 0 mM and 25 mM glucose cultured H9c2 cells. Hence, we addressed the functional roles of myogenin when H9c2 cells were challenged by palmitic acid via the knockdown of the myogenin gene. We screened the most efficient effect of downregulation of the myogenin gene from seven short-hairpin *myogenin* (*shMyogenin*) constructs in H9c2 cells (Figure 4A). We reconfirmed the silencing effect on the *myogenin* gene via its mRNA and protein levels (Figure 4B,C). The basal mRNA levels affected by myogenin were those of *COX-2* and *p53*, and the basal protein levels affected by myogenin were those of COX-2, p21, and histone H3. The response to palmitic acid was affected by myogenin for *COX-2* mRNA and protein. We further examined the functional role of myogenin in H9c2 cells cultured with relatively lower glucose concentrations (Figure 5). The effects of palmitic acid on cyclin D1, H3, and γH2A.x were highlighted in *myogenin*-silenced H9c2 cells. The downregulation of p53 and upregulation of ATF3 by palmitic acid was observed in H9c2 cells treated with 2.5 mM and 5.56 mM glucose, regardless of *myogenin* expression.

**Figure 6:**
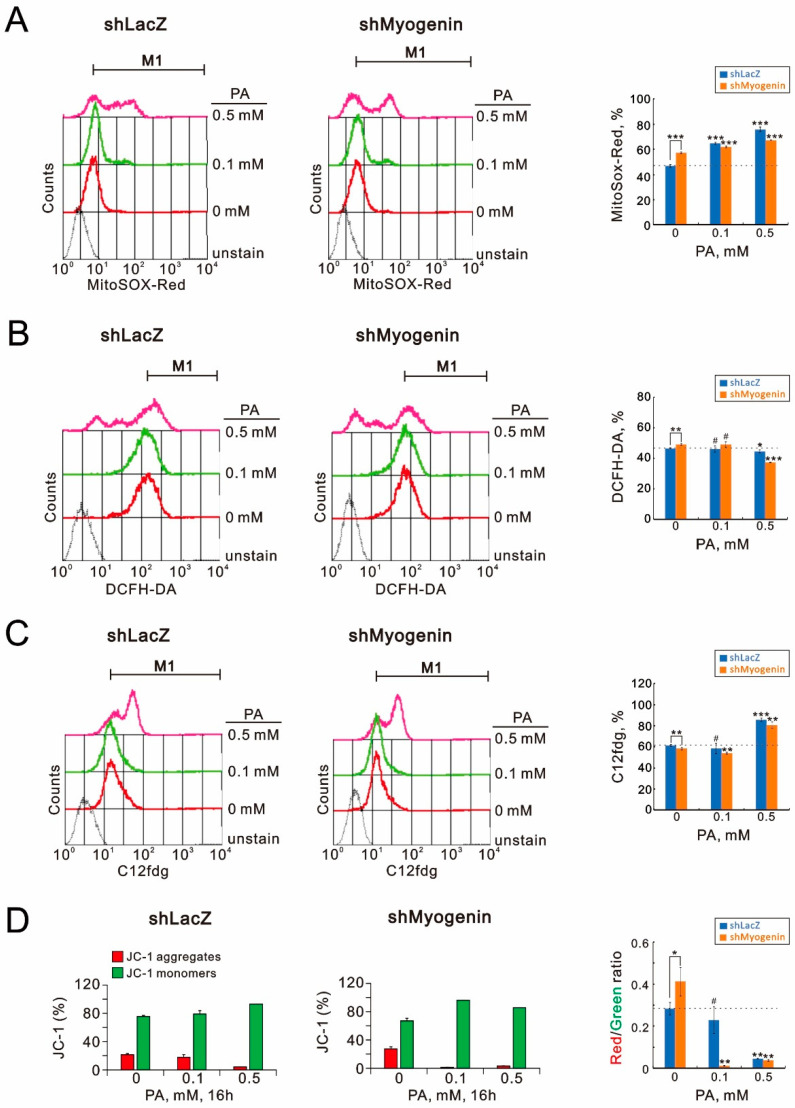
The effects of *myogenin* gene silencing combined with palmitic acid on mitochondrial ROS generation, cytosolic ROS generation, SA-β-Gal activity, and the mitochondrial membrane potential expression profile in H9c2 cells. The *shLacZ* and *shMyogenin* H9c2 cells were treated with the indicated concentrations of palmitic acid (0, 0.1, 0.5 mM) for 16 h. (**A**) The live cells were stained with 5 μM MitoSOX^TM^ Red for 15–20 min at 37 °C and subjected to flow cytometry analysis. (**B**) Live cells were incubated with 10 μM DCFH-DA in the dark at 37 °C for 30 min and then harvested were assayed using a flow cytometer. (**C**) After the incubation, the cells were harvested and stained with 33 μM C_12_FDG for 15–20 min at room temperature. Fluorescence intensity was then evaluated using a FACSCalibur flow cytometer. (**D**) All dead and viable cells were harvested and stained with 1× binding buffer containing the MMP-sensitive fluorescent dye JC-1 for 30 min at 37 °C in the dark. After washing the cells with 1× binding buffer, JC-1 fluorescence was analyzed on channels FL-1 and FL-2 of the FACSCalibur flow cytometer. The bars represent the mean ± SD of three independent experiments. Statistical significance is indicated by # for *p* > 0.05, * for *p* < 0.05, ** for *p* < 0.01, and *** for *p* < 0.001, determined using Student’s *t*-tests.

The inductions of ROS and cellular senescence and the disruption of mitochondrial membrane potentials by palmitic acid were addressed regardless of the involvement of myogenin [32,33]. We analyzed the effects of palmitic acid and myogenin on mitochondrial and cytosolic ROS generation, cellular senescence, and mitochondrial membrane potential via flow cytometry analysis with MitoSox, DCFH-DA, C12fdg, and JC-1 dyes, respectively (Figure 6). In Figure 6A, our data show that myogenin repressed mitochondrial ROS generation in controls, whereas it had a positive role in palmitate-induced mitochondrial ROS generation in H9c2 cells. In Figure 6B, our data show that myogenin had a negative effect on cytosolic ROS generation in controls, but the suppression of ROS generation by palmitic acid was induced in *shMyogenin*-H9c2 cells. Myogenin was found to play positive roles in cellular senescence and palmitic-acid-induced cellular senescence in H9c2 cells (Figure 6C). The treatment of the *shLacZ* control resulted in the disruption of mitochondrial membrane potential in H9c2 cells. The knockdown of *myogenin* (*shMyogenin*) expression resulted in a greater likelihood of normal mitochondrial membrane potential (JC-1 aggregates) in H9c2 cells compared to the *shLacZ* controls, whereas we observed a greater likelihood of normal mitochondrial membrane potential (JC-1 aggregates and JC-1 monomers) in 0.1 mM palmitic-acid-treated *shMyogenin*-H9c2 cells (Figure 6D). Our current data suggest that myogenin might be involved in mitochondrial and cytosolic ROS generation, cellular senescence, and mitochondrial membrane potential in H9c2 cells; however, myogenin might have different functional roles because of the effects of palmitic acid on the abovementioned cellular functions.

### 2.3. Evaluating the Clinical Role of Myogenin in Myocardial Tissue: Insights from GSE150059 Myocardial Tissue Sample Database

**Figure 7:**
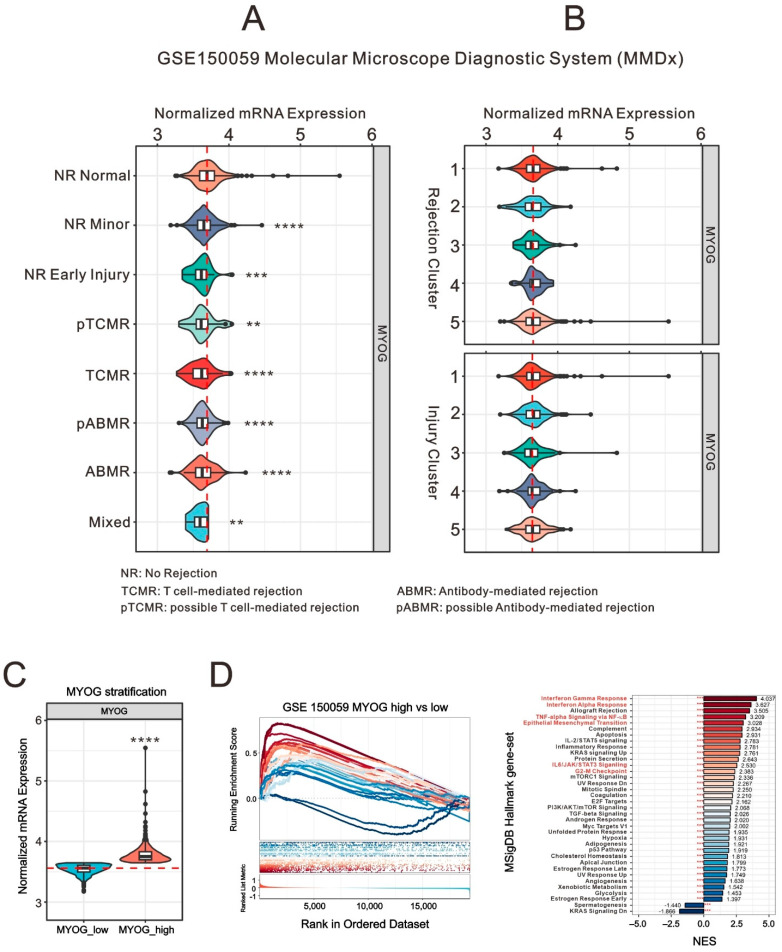
Clinical implications of *myogenin* (*MYOG*) in myocardial tissues through GSE150059 analysis. (**A**,**B**) Violin plots showcasing the differential expression of the *MYOG* gene based on the myocardial injury diagnosis and clustering provided by the GSE150059 dataset. Statistical differences between the topmost group and all other groups in each graph were determined using Student’s *t*-test. Symbols denote levels of significance: ** *p* < 0.01, *** *p* < 0.001, **** *p* < 0.0001. (**C**) Violin plot presenting the distribution of *MYOG* expression based on Z-score categorization. Statistical difference between *MYOG*_low and *MYOG*_high was determined using Student’s *t*-test. **** *p* < 0.0001. (**D**) The GSEA plot depicts the enrichment trend of Hallmark gene sets. Red to blue lines represent the enrichment scores, sorted from high to low by NES, with the scores labeled at the end of the NES bars. Red asterisks represent FDR values, with *** indicating FDR < 0.01.

In order to comprehensively assess the role of myogenin in clinical myocardial tissue, we employed the GSE150059 myocardial tissue sample database. This database incorporates 1320 prospective biopsy samples from 645 patients across 13 international centers, 34,548,198 in total. With several transcriptomic fractions related to injury utilized as inputs, molecular damage was evaluated through prototype analysis. The molecular scores assigned using the Molecular Microscope Diagnostic System (MMDx)—Heart provide precision, accuracy, and standardization in heart transplant diagnostics that can be improved by incorporating central molecular biopsy assessments in the measurement of rejection and injury changes [34]. Our examination of *myogenin* expression followed the classification provided by the literature under different clinical conditions, including no rejection (NR) normal, NR minor, NR early injury, T-cell-mediated rejection (TCMR), antibody-mediated rejection (ABMR), mixed, and possible rejection (pABMR and pTCMR). The minor category is characterized by low-level inflammation in non-rejecting biopsies. Figure 7A illustrates a significant reduction in *myogenin* expression in myocardial tissues of varying injury degrees compared to normal tissues (NR normal). Injury is universal and programmed into the process of all organ transplants, including the stress of donation, preservation, and implantation “wounds”; additionally, the graft tissue evokes a response to wounding. Rejection-associated transcript-based analysis also identified an inflamed “early injury” state distinct from rejection that evokes inflammation and shares mechanisms with injury. However, no significant difference in *myogenin* expression was observed within the additional rejection and injury clusters documented in the literature (Figure 7B). Further, we normalized *myogenin* expression across all samples, categorizing samples with a Z-score greater than or equal to zero as *MYOG*_high and those with a Z-score less than zero as *MYOG*_low and performed further analysis using the Molecular Signatures Database (MSigDB) (Figure 7C). Conducting a GSEA stratified by *myogenin* expression and using the Hallmark gene set for the preliminary analysis of 50 major biological responses (Figure 7D) revealed that *MYOG*_high is associated with numerous immune responses. These include the interferon gamma response, interferon alpha response, TNF-alpha signaling via NF-κB, and IL6/JAK/STAT3 signaling. It may also influence the epithelial–mesenchymal transition and G2-M checkpoint of myocardial cells, corroborating our earlier research findings. This study provides important insights into the role of myogenin in the pathological processes of myocardial injury.

### 2.4. Unraveling the Complexities of Fatty-Acid-Related Gene Expression in Myogenin-High Cardiovascular Tissue

**Figure 8:**
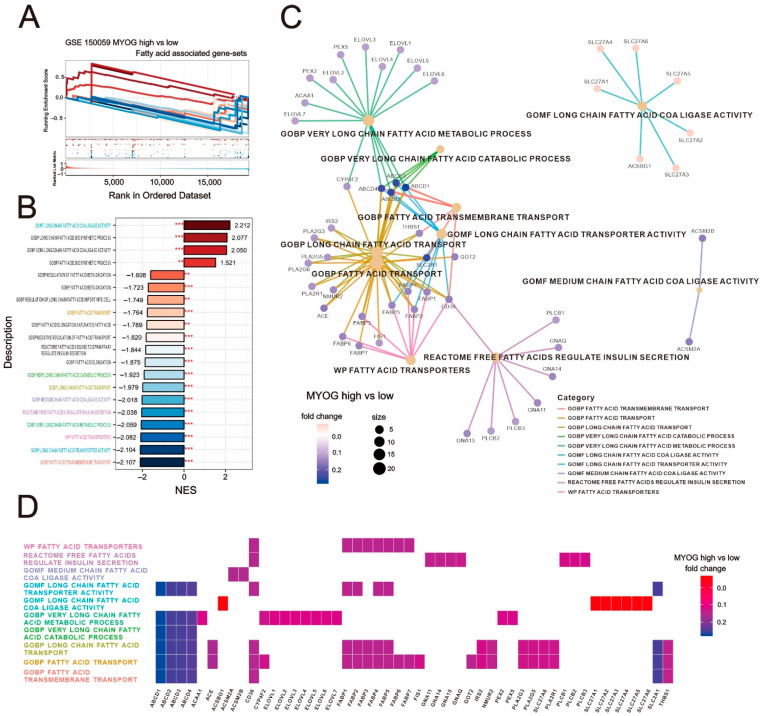
Decoding fatty acid genes in *myogenin*-rich cardiovascular tissue. (**A**,**B**) The GSEA plots show the enrichment trend in fatty-acid-related gene sets. Red to blue lines represent enrichment scores, sorted from high to low by NES, with scores labeled at the end of the NES bars. Red asterisks represent FDR values: ** FDR < 0.05, *** FDR < 0.01. (**C**) The cnetplot shows the top ten fatty-acid-associated gene sets with the lowest FDR. The size indicates the number of top genes associated with the gene set, while the fold change indicates the difference in expression (log2) between the high- and low-*MYOG* groups. (**D**) The heat map organizes all associated genes from the cnetplot and presents them in alphabetical order.

Considering the potential impact of fatty acids on *myogenin*, we selected gene sets related to fatty acids from Gene Ontology (GO) and analyzed the enrichment scores of these fatty-acid-related gene sets using GSEA (Figure 8A). Figure 8B outlines the normalized enrichment scores (NESs) for various fatty-acid-related gene sets, revealing that in clinical myocardial tissue, GOMF (Gene Ontology Molecular Function) long-chain fatty acid CoA ligase activity is the most significantly positively enriched gene set in *MYOG*_high samples. Conversely, multiple gene sets associated with fatty acid transport and metabolism, including GOBP (Gene Ontology Biological Pathway) fatty acid transport, GOBP very-long-chain fatty acid catabolic process, GOBP long-chain fatty acid transport, GOMF medium-chain fatty acid CoA ligase activity, GOBP very-long-chain fatty acid metabolic process, WP fatty acid transporters, GOMF long-chain fatty acid transporter activity, and GOBP fatty acid transmembrane transport, were all significantly negatively correlated (Figure 8B). Using clusterProfiler’s cnetplot analysis of the leading-edge gene distribution of the negatively enriched gene sets, we found that *MYOG*_high is related to the expression of specific gene families (Figure 8C). For instance, the *SLC27A* family was significantly upregulated, contributing to the positive enrichment of GOMF long-chain fatty acid CoA ligase activity. Meanwhile, families such as *ELOVL*, *ABCD*, *PLA2G*, and *FABP* were significantly downregulated, leading to the negative enrichment of various fatty-acid-transport-related gene sets. The expression and distribution of related gene families can be observed in the heat map provided in Figure 8D. This finding provides valuable insight into the differential role of fatty-acid-associated gene expression in cardiovascular tissues expressing elevated levels of *myogenin*.

**Figure 9:**
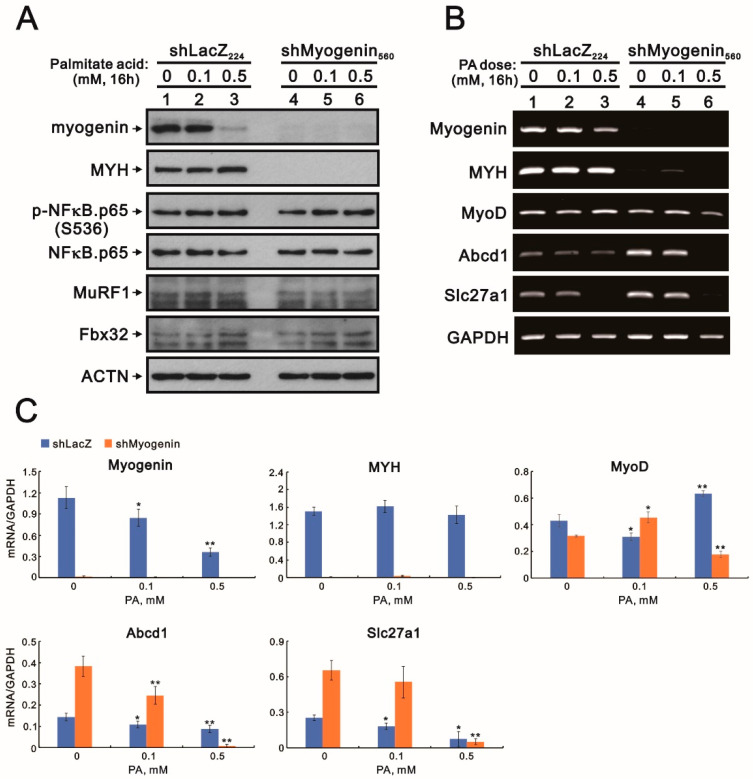
The cell signaling effects of *shLacZ*/*shMyogenin*-H9c2 cells with palmitic acid treatment on proteins and the mRNA gene expression profile. The *shLacZ* and *shMyogenin* H9c2 cells were treated with the indicated concentrations of palmitic acid (0, 0.1, 0.5 mM) for 16 h. (**A**) Cell lysates were subjected to Western blotting analysis. ACTN was used as a loading control protein. (**B**) Total RNA was subjected to RT-PCR analysis. *GAPDH* was used as a loading control. (**C**) The mRNA bands were quantified through pixel density scanning and evaluated using Image J, version 1.44a (http://imagej.nih.gov/ij/) (accessed on 13 August 2023). The ratios of mRNA/*GAPDH* are listed in the H9c2 cells. Bars depict the mean ± SD of three independent experiments. Student’s *t*-tests were analyzed and compared various concentrations of palmitic acid with vehicle in H9c2 *shLacZ* and *shMyogenin* cells. * *p* < 0.05 and ** *p* < 0.01.

NF-κB might be a therapeutic target for muscular dystrophy because of the direct induction of muscle atrophy and the transcription of atrogin-1 and MuRF1 for protein degradation [20,28]. Figure 9A (Western blotting data) shows that NF-κB and two muscle E3 ligase proteins, MuRF1 and Fbx32, were not affected by palmitic acid treatment or the downregulation of myogenin in H9c2 cells compared with the suppression of myogenin by palmitic acid treatment and the induction of *MHY* (*myosin heavy chain*) by myogenin. We further focused on and verified *Abcd1* and *Slc27a1* genes in shMogyenin-H9c2 cells treated with palmitic acid to support the functional role of myogenin in heart transplantation. Our data show that the expression levels of *Abcd1* and *Slc27a1* genes, as well as the myogenin gene, were downregulated by palmitic acid in the H9c2 cells (Figure 9B). Our data suggest that myogenin might be a repressor of *Abcd1* and *Slc27a1* genes, an activator of the *MHY* gene, and neutral factor of the *MyoD* gene in H9c2 cells. High-concentration palmitic acid (0.5 mM) still suppressed the expression of *Abcd1* and *Slc27a1* genes in *shLacZ* and *shMogyenin*-H9c2 cells (Figure 9C). The negative or positive effect of palmitic acid on *MyoD* gene expression was dependent on its concentration in *shLacZ* and *shMogyenin*-H9c2 cells (Figure 9C).

## 3. Discussion

Lipid-deposition-induced comparative cardiac lipotoxicity is one of the pathological alterations that contribute to cardiac aging in the failing heart [1,5]. Cardiac lipotoxicity plays a pathological role in the development of obesity-induced cardiovascular diseases [2]. Metabolic-syndrome-associated cardiac diseases increase the risk of developing HF. Sarcopenia indicates an age-related loss of skeletal muscle, and the prevalence of sarcopenia in chronic HF patients is up to 20% and may progress into cardiac cachexia [35]. HF and sarcopenia could benefit from a common therapeutic approach based on many similar pathogenetic pathways. The reversal of muscle cell differentiation and the creation of mononucleated cells could be found by the downregulation of myogenin proteins in muscle cells [20]. A recent study demonstrated that myogenin is an essential transcription factor for adult myofiber growth and muscle stem cell homeostasis [36]. Hence, it is an important issue to understand the regulation of myogenin expression and functions in the response to exposure to varied stresses. In this study, we first examined and verified the cytotoxic effect of palmitic acid on H9c2 cells. The reduction in *myogenin* mRNA and protein expression by palmitic acid was independent of the effect of glucose. Meanwhile, the induction of *COX-2* and *ATF3* mRNAs and proteins by palmitic acid were dependent on the presence of glucose. In addition, palmitic acid failed to disrupt cell cycle progression when H9c2 cells were not treated with glucose. Next, our data show that myogenin was a repressor for mitochondrial and cytosolic ROS generation, whereas myogenin had a positive role in palmitate-induced mitochondrial and cytosolic ROS generation in H9c2 cells. The differential functional roles of myogenin in cellular senescence and mitochondrial membrane potential might be related to the generation of ROS in H9c2 cells. Finally, we correlated the expression of myogenin with clinical myocardial tissue via a GSE150059 myocardial tissue sample database. With these results, we provide some insight into the functional role of myogenin in lipid- and glucose-induced cardiac cell stresses. Our work also sheds light on the understanding of myogenic factors involved in aging heart functions.

There are many transcription factors, including Foxo proteins and myogenin, that regulate the expression of MuRF1 and atrogin-1 to control protein degradation and muscle atrophy [28]. Myogenin may be a specific regulator of adaptive responses and Foxo1 and 3a may primarily regulate other processes such as antioxidant response to avoid pathological hypertrophy in the heart [29]. The release of inflammatory factors can activate the NF-κB pathway, suggesting that NF-κB might be a therapeutic target for muscular dystrophy because of the direct induction of muscle atrophy and the transcription of atrogin-1 and MuRF1 for protein degradation, which not only directly induces muscle atrophy but also mediates the transcription of atrogin-1 and MuRF1, leading to increased protein degradation, which indicates that NF-κB is expected to become a therapeutic target for muscular dystrophy [20,28]. Our current analysis of MSigDB Hallmark gene sets correlates inflammation-related pathways with myogenin. However, our Western blotting data show no effect on NF-κB, MuRF1, or Fbx32 protein expression by myogenin in H9c2 cells. These two inconsistent results reveal differential expression of target mRNAs and proteins, suggesting that complicated regulatory mechanisms need to be further addressed.

Twist, *miR-186*, HuR, CACNb1, p53, and the MyoD/FoxO3 complex may mediate myogenin expression through different pathways [22,23,24,26,27,37,38]. TNF-α, a cytokine that commonly increases during HF, can suppress the expression of *myogenin* in differentiating myocytes [39]. A recent study demonstrated that H_2_O_2_, but not TNF-α markedly suppressed the expression of *miR-328* in gastric cancer cells [40]. Combined with these findings, the suppression of *myogenin* by TNF-α depends on the cell type. A working model was proposed for a repressor of *myogenin* gene regulation by downregulating *miR-328* expression and increasing *CACNb1* expression to relieve the doxorubicin-associated myocardial toxicity in H9c2 cells [27]. Hence, the positive and negative regulatory mechanisms of *myogenin* expression by glucose and palmitic acid, respectively, remain to be further investigated in H9c2 cells.

The aged myoblasts showed that increased atrophy and inflammatory factors diminish differentiation capacity and disrupt myogenic lineage via myogenin [41]. Mitochondria are the major organelles for ROS generation and might be responsible for the cytosolic ROS generation via the oxidation–reduction system between mitochondria and cytosol in cells [42]. Mitochondrial ROS has been reported to be involved in the promotion of longevity as a signaling molecule to negatively modulate the excessive immune responses by ROS [43]. It is well known that NF-κB is the most important transcription factor in the immune system and functions as a negative regulator of myogenesis via the suppression of myogenic gene expression, such as *MyoD* [41]. In addition, mitochondrial biogenesis, including mass and mtDNA copy number, and mitochondrial respiration are markedly increased during the progression of myogenic differentiation [44]. Compared with our current findings of myogenin in cellular senescence and mitochondrial functions, myogenin is the major terminal muscle differentiation transcription factor and its amounts were downregulated by palmitic acid in this study. It is an interesting issue to address whether targets of myogenin involve the cellular senescence and mitochondrial functions and/or myogenin directly interact with other transcription factors, such as NF-κB, for the regulation of cellular functions, including senescence, ROS generation, mitochondrial functions in cells.

The exit from the cell cycle and the attenuation of cardiac muscle characteristics via the blunting in expression of a cardiac-specific subunit of the L-type Ca^2+^ channel is the entire skeletal muscle differentiation process of H9c2 cardiomyoblasts. The skeletal muscle differentiation induced by serum withdrawal in H9c2 cells is mediated through a progressive decrease in intracellular cAMP content. ATF3 is a member of the cAMP response element-binding protein family [45]. ATF3 is a transcription factor for vital physiological processes, including apoptosis, glucose metabolism, lipid metabolism, and inflammation, and highly regulated by glucose and palmitic acid in this study [46]. However, our downregulation of *myogenin* failed to induce an observed disruption in *ATF3* mRNA expression in this study. Hence, it is important to find out how to drive the differentiation of a multipotent cell towards a specific cell type via the use of myogenin in a precise muscle differentiation step and provide therapeutic solutions with new targets.

Compared with conventional assessments, the precise measurements of molecular platforms provide an opportunity to improve the assessment of heart transplant endomyocardial biopsies with mechanistic insights. MMDx—Heart was developed to diagnose molecular TCMR and ABMR based on expression of rejection-associated transcripts to distinguish them from other forms of inflammation for heart transplant endomyocardial biopsies [34]. The application of injury-associated transcript sets to assess the degree of parenchymal injury and atrophy fibrosis for the effect of rejection on the parenchyma in MMDx—Heart. The short-term risk of HF is strongly determined by parenchymal injury via the analysis of TCMR and ABMR. TCMR directly injures the parenchyma and ABMR usually induces microcirculation stress. The long-term impact of minor NR changes in late heart transplant syndromes, such as atrophy fibrosis, needs to be verified. Our current analytic data show that the expression levels of *myogenin* in the studied population were below the average level of normal NR. In addition, the expression level of *myogenin* was maximally upregulated by 2.5 mM glucose, and 0.5 mM palmitic acid suppressed *myogenin* expression in no or high concentrations of glucose in H9c2 cell treatment. Hence, the lower-than-normal expression level of *myogenin* in response to palmitic acid might represent a potential risk for NR minor, NR early injury, TCMR, ABMR, and mixed heart transplantation outcomes. However, complicated lipid metabolism and the question of whether the relevant target genes are regulated by myogenin also highlight the usefulness of a platform such as MMDx—Heart, which could help elucidate the functional role of myogenin in heart transplantation.

## 4. Materials and Methods

### 4.1. Cell Culture and Chemicals

H9c2 cells were cultured in Dulbecco’s modified Eagle’s medium supplemented with 10% fetal bovine serum and 1% penicillin–streptomycin (Invitrogen; Waltham, MA, USA). Glucose, palmitic acid, 2′,7-dichlorofluorescein diacetate (DCFH-DA), propidium iodide (PI), and thiazolyl blue tetrazolium bromide (MTT) were purchased from Sigma-Aldrich (St. Louis, MO, USA). MitoSOX Red was obtained from Invitrogen.

### 4.2. Western Blot Analysis

The cell lysates were prepared in lysis buffer (100 mM Tris-HCl, pH 8.0, 150 mM NaCl, 0.1% SDS, and 1% Triton X-100) at 4 °C. The extractions were separated via SDS-PAGE, transferred onto a polyvinylidine difluoride membrane (Millipore, Bedford, MA, USA), and detected using antibodies against H3, p-NFκB.p65 (S536) (Cell Signaling Technology, Danvers, MA, USA), α-actinin (ACTN), p53, p21, Cyclin D1, H3P, MYH, Twist, COX-2, ATF-3, NFκB-p65 (Santa Cruz Biotechnology, Santa Cruz, CA, USA), γH2A.X, myogenin, MuRF1, and Fbx32 (Abcam, Cambridge, UK). The membranes were incubated first with primary antibodies against proteins of interest and then with HRP-conjugated secondary antibodies (anti-mouse IgG, AP192P and anti-rabbit IgG, AP132P, Merck-Millipore, Burlington, MA, USA). The immunoreactive proteins were detected using ECL^TM^ Western Blotting Detection Reagent and Amersham Hyperfilm^TM^ ECL (GE Healthcare, Waukesha, WI, USA). The procedural details have been described in our previous publications [47,48].

### 4.3. Reverse Transcription–Polymerase Chain Reaction (RT-PCR)

H9c2 cells were lysed in TRIzol reagent (Invitrogen) to isolate total RNAs. One microgram RNA was subjected to reverse transcription for first strand cDNA synthesis using MMLV reverse transcriptase for 60 min at 37 °C (Epicentre Biotechnologies, Madison, MI, USA). The PCR reactions were run on a Veriti^TM^ Simpli Thermal Cycler (Applied Biosystems, Carlsbad, CA, USA) for further agarose gel analysis. The procedural details have been described in our previous publications [47,48]. The mRNA bands were quantified through pixel density scanning and evaluated using Image J, version 1.44a (http://imagej.nih.gov/ij/, accessed on 13 August 2023). All PCR primer sequences are shown in Table 1.

### 4.4. Fluorescence-Activated Cell Sorting (FACS) Analysis

For cell cycle evaluation, the cells were fixed in 70% ice-cold ethanol and stored at −30 °C overnight, after which they were washed twice with ice-cold PBS supplemented with 1% FBS and stained with PI solution (5 μg/mL PI in PBS, 1% Tween 20, and 0.5 μg/mL RNase A) for 30 min at 37 °C in the dark. The FACS analysis was based on the measurement of the cellular DNA content of nuclei labeled with propidium iodide (PI). The cells were then subjected to FACS, and cell cycle analysis was performed using a FACSCalibur flow cytometer and Cell Quest Pro software (BD Biosciences, Franklin Lakes, NJ, USA) as previously described [47,48].

The intracellular ROS levels were determined using the fluorescent marker DCFH-DA. Briefly, the cells were treated under the selected conditions and then washed twice with PBS and incubated with 10 μM DCFH-DA in the dark at 37 °C for 30 min, after which they were harvested. Afterwards, the cells were washed once with PBS, and then the DCFH-DA fluorescence intensity was analyzed on the FL-1 channel of the FACSCalibur flow cytometer using Cell Quest Pro Software (BD Biosciences). The procedural details have been described in our previous publications [47,48].

For the flow cytometric senescence assays, senescence-associated β-galactosidase (SA-β-Gal) activity was measured using the fluorescent substrate 5-dodecanoyl-aminofluorescein di-β-D-galactopyranoside (C_12_FDG) (Invitrogen) according to the manufacturer’s instructions. Briefly, the cells were plated on 6-well culture plates and treated with the selected conditions. After the incubation, the cells were harvested, washed twice with PBS, and stained with 33 μM C_12_FDG for 15–20 min at room temperature. The fluorescence intensity was then evaluated using a FACSCalibur flow cytometer and Cell Quest Pro software (BD Biosciences). The procedural details have been described in our previous publications [47,48].

For determining the mitochondrial ROS level, the fluorescent marker MitoSOX^TM^ Red (Invitrogen, M36008) was used. MitoSOX^TM^ Red mitochondrial superoxide indicator is a fluorogenic dye for the highly selective detection of superoxide in the mitochondria of live cells. Once in the mitochondria, MitoSOX^TM^ Red reagent is oxidized by the superoxide and exhibits red fluorescence. Briefly, the cells were plated on 6-well culture plates and treated under the selected conditions. The living cells were then stained with 5 μM MitoSOX^TM^ Red for 15–20 min at 37 °C and harvested. After washing the cells once with PBS, the fluorescence intensity was then evaluated using a FACSCalibur flow cytometer and Cell Quest Pro software (BD Biosciences). The procedural details have been described in our previous publications [47,48].

For mitochondrial membrane potential (MMP) analysis, mitochondrial depolarization was measured as a function of the decrease in the red/green fluorescence intensity ratio. All dead and viable cells were harvested, washed with PBS, and stained with 1× binding buffer containing the MMP-sensitive fluorescent dye JC-1 (BD^TM^ MitoScreen, Mitochondrial Membrane Potential Detection JC-1 Kit) for 30 min at 37 °C in the dark. After washing the cells with 1× binding buffer, the JC-1 fluorescence was analyzed on channels FL-1 and FL-2 of the FACSCalibur flow cytometer and Cell Quest Pro software (BD Biosciences) to detect the monomer (green fluorescence) and aggregate (red fluorescence) forms of the dye, respectively. The procedural details have been described in our previous publications [49].

### 4.5. Gene Silencing

Our *shLacZ*- and *shMyogenin*-H9c2 cells were constructed and screened via seven shRNA lentiviral vectors targeting mouse *myogenin* (Clone ID: TRCN0000081561) for the screening of rat *myogenin* and a pLKO control lenti-viral vector (Clone ID: TRCN000072224) for the *LacZ* gene purchased from the National RNAi Core Facility, Academia Sinica, Taiwan. The H9c2 cells infected with these lentiviruses containing the target gene shRNAs were selected in 2 μg/mL puromycin. All pooled populations of knockdown cells were verified by Western blotting analysis and applied for this study. Procedural details were described previously [27].

### 4.6. Gene Set Enrichment Analysis (GSEA) of MYOG Expression and Fatty-Acid-Related Genes in GSE150059 Samples

Gene Set Enrichment Analysis (GSEA) was performed using the clusterProfiler package [50]. The raw gene expression matrices and phenotype data for GSE150059 samples were retrieved from the GEO database using the GEOquery package. The differences in *MYOG* expression among NR Normal, Rejection Cluster 1, and Injury Cluster 1, as well as between these groups and all other categories, were analyzed using a Student’s *t*-test approach. *MYOG* expression across all samples was standardized to Z-scores. Samples with Z-scores equal to or greater than zero were categorized as *MYOG*_high, while those with Z-scores less than zero were classified as *MYOG*_low. To carry out the GSEA, we first downloaded the Hallmark gene set from the Molecular Signatures Database (MsigDB) website [51]. For the fatty-acid-related gene set, we downloaded MsigDB C2 curated gene sets and C5 Gene Ontology sets, filtered them with “fatty acid” as the keyword to create a file in the Gene Matrix Transposed (GMT) file format, and then analyzed them using the gseaplot2 function for plotting. To explore the relationship and distribution of the leading-edge genes in the top 10 gene sets with the smallest false discovery rate (FDR), we utilized the cnetplot and heatmap functions from the clusterProfiler package.

### 4.7. Statistical Analysis

The values are expressed as the mean ± SD of at least three independent experiments. All comparisons between groups were made using Student’s *t*-tests. The statistical significance was set at *p* < 0.05.

## 5. Conclusions

In this study, the reduction in *myogenin* mRNA and protein expressions by palmitic acid was independent of the effect of glucose. Cell cycle progression failed to be disrupted by palmitic acid when H9c2 cells were in the no-glucose condition. Myogenin may be involved in palmitic-acid-induced mitochondrial and cytosolic ROS generation, cellular senescence, and mitochondrial membrane potential. Our data show the complexities of fatty-acid-related gene expression in myogenin-high cardiovascular tissue. Combined with these results, we might elucidate the functional role of myogenin in lipid- and glucose-induced cardiac cell stresses, providing valuable insight into the differential role of fatty-acid-associated gene expression in cardiovascular tissues.

## Figures and Tables

**Table 1:** Primers used in this study.

Gene Name	Primer Sequence (Forward)	Primer Sequence (Reverse)
*Troponin I*	5′-GCAAAAGTCACCAAGAACATC-3′	5′-GCGCCAGTCTCCCACCTCCCGG-3′
*Myogenin*	5′-CTACCTTCCTGTCCACCTTC-3′	5′-CTCCAGTGCATTGCCCCACT-3′
*MYH*	5′GCCTCATCCACACCAAGAAGA-3′	5′-TCCACCAGATCCTGCAATCTC-3′
*Myo D1*	5′-CAGCGGGCACCACCAG-3′	5′-ATGCTGGACAGGCAGTC-3′
*p53*	5′-GCAACTATGGCTTCCACCTG-3′	5′-CACGAACCTCAAAGCTGTCC-3′
*p21*	5′-GCTGTCTCCAGGAGGCCCG-3′	5′-GCTGGTCTGCCTCCGTTTTCG-3′
*Cyclin D1*	5′-ATGGAACACCAGCTCCTGTG-3′	5′-CTTAGAGGCCACGAACATGC-3′
*ATF3*	5′-GAGGATTTTGCTAACCTGAC-3′	5′-TAGCTCTGCAATGTTCCTTC-3′
*COX-2*	5′-GTCTCTCATCTGCAATAATGTG-3′	5′-ATCTGTGTGGGTACAAATTTG-3′
*FASN*	5′-TGAGCCTCATGCGCCTGGAC-3′	5′-CGCACCTCCTTGGCAAACAC-3′
*Abcd1*	5′-TCCTGTCTGTGTATGTTGCCC-3′	5′-GGAGAGAAGGCTCGAAGCAC-3′
*Slc27a1*	5′-CAAGGTCAACGAGGACACGA-3′	5′-ACAGCCACTCCATACACAGC-3′
*GAPDH*	5′-CTTCATTGACCTCAACTAC-3′	5′-GCCATCCACAGTCTTCTG-3′

## Data Availability

Not applicable.
